# Increased risk of acute kidney injury in coronavirus disease patients with renin–angiotensin–aldosterone-system blockade use: a systematic review and meta-analysis

**DOI:** 10.1038/s41598-021-92323-8

**Published:** 2021-06-30

**Authors:** Sul A Lee, Robin Park, Ji Hyun Yang, In Kyung Min, Jung Tak Park, Seung Hyeok Han, Shin-Wook Kang, Tae-Hyun Yoo

**Affiliations:** 1 Department of Internal Medicine, College of Medicine, Institute of Kidney Disease Research, Yonsei University, 50-1 Yonsei-ro, Sinchon-dong, Seodaemun-gu, Seoul, South Korea; 2 Department of Medicine, MetroWest Medical Center/Tufts University School of Medicine, Framingham, MA USA; 3 Biostatistics Collaboration Unit, Department of Biomedical Systems Informatics, Yonsei University College of Medicine, Seoul, South Korea

**Keywords:** Kidney diseases, Viral infection

## Abstract

Acute kidney injury (AKI) is a severe complication of coronavirus disease (COVID-19) that negatively affects its outcome. Concern had been raised about the potential effect of renin–angiotensin–aldosterone system (RAAS) blockades on renal outcomes in COVID-19 patients. However, the association between RAAS blockade use and incident AKI in COVID-19 patients has not been fully understood. We investigated the association between RAAS blockade exposure and COVID-19-related AKI in hospitalized patients through meta-analysis. Electronic databases were searched up to 24th December 2020. Summary estimates of pooled odds ratio (OR) of COVID-19-related AKI depending on RAAS blockade exposure were obtained through random-effects model. The random-effect meta-analysis on fourteen studies (17,876 patients) showed that RAAS blockade use was significantly associated with increased risk of incident AKI in hospitalized COVID-19 patients (OR 1.68; 95% confidence interval 1.19–2.36). Additional analysis showed that the association of RAAS blockade use on COVID-19-related AKI remains significant even after stratification by drug class and AKI severity. RAAS blockade use is significantly associated with the incident AKI in hospitalized COVID-19 patients. Therefore, careful monitoring of renal complications is recommended for COVID-19 patients with recent RAAS blockade use due to the potential risk of AKI.

## Introduction

Coronavirus disease (COVID-19) is a protean viral illness with a potentially life-threatening course, that has caused unforeseen and unprecedented socioeconomic burdens. The clinical course of COVID-19 varies widely from asymptomatic infection to deadly severe acute respiratory distress syndrome, multi-organ failures, and death^1^. To date, around 3.2 M people have died due to severe acute respiratory syndrome coronavirus 2 (SARS-CoV-2) infection worldwide, and almost 20% of the total deaths have occurred in the United States. (https://www.who.int/emergencies/diseases/novel-coronavirus-2019). Despite extensive global efforts to identify potential therapeutics for COVID-19 patients^2–5^, the vast majority have not resulted in clinically meaningful improvement. While waiting for the worldwide vaccination and the establishment of herd immunity, and with a limited therapeutic armamentarium, a better understanding of the risk factors associated with severe/fatal COVID-19 course and meticulous medical attention to mitigate these risk factors are crucial in improving its clinical outcome.

Acute kidney injury (AKI) is one of the most common complications of COVID-19, which has been found to be significantly related to poorer clinical outcome and increased risk of mortality in COVID-19 patients^6^. The pathogenesis of AKI in COVID-19 patients is likely multifactorial, including direct injury through viral tropism leading to endothelial dysfunction, coagulopathy, and complement activation as well as indirect injury through organ crosstalk, dehydration, and exposure to nephrotoxins while receiving COVID-19 treatment^7^. Several risk factors for COVID-19-induced AKI have been addressed in recent studies including older age, male sex, severity of COVID-19, and the levels of ferritin, creatine kinase, and brain natriuretic peptide^8,9^. Notably, concern had been raised about the use of renin–angiotensin–aldosterone system (RAAS) blockade and its potential risk on incident AKI in COVID-19 patients based on its potential effect on the reduction of glomerular filtration rate as well as increased susceptibility and severity of COVID-19 by enhancing angiotensin-converting enzyme 2 (ACE2) which is a functional receptor protein for SARS-CoV-2^7^.

RAAS inhibitors are known to affect ACE2 expression and activity, which can hypothetically increase the risk of SARS-CoV2 infection by facilitating viral entry^10^. On the other hand, an alternative hypothesis has suggested that RAAS blockade may mitigate viral entry into cells by restoring ACE1/ACE2 imbalance^11^. Beyond these speculative assumptions, several clinical studies have shown no clear association between RAAS inhibitor use and the incidence of COVID-19 or mortality^12–15^. However, the safety data regarding RAAS blockade use on COVID-19-related renal dysfunction are relatively scarce. Several recent studies have shown the potential detrimental effects of angiotensin-converting-enzyme inhibitor (ACEI) and angiotensin receptor blocker (ARB) use on the development of AKI in COVID-19 patients^16–18^, but these studies have limitations due to the small number of participants.

ACEIs and ARBs are widely used antihypertensive medications, and their use is most prevalent in the elderly and chronic kidney disease patients, who belong to the highest risk groups for the development of COVID-19-related mortalities and multiorgan failure. Thus, further investigation of the potential association of RAAS blockade exposure with the development of AKI in COVID-19 patients is crucial to provide proper guidance to healthcare providers in order to prevent unnecessary exposure or discontinuation of RAAS inhibitors in COVID-19 patients. Therefore, we conducted a systematic review and meta-analysis of the association between RAAS blockade use and AKI development in laboratory-confirmed or clinically diagnosed COVID-19 patients.

## Methods

### Search strategy and study selection

Published articles reporting incident AKI in COVID-19 patients and their use of ACEIs or ARBs from January 1, 2020 to December 24, 2020 were identified by searching PubMed, Embase, Cochrane, and Scopus. A search update was conducted on January 15, 2021. An example search strategy used for PubMed was as follows: (COVID-19 OR SARS-CoV-2 OR coronavirus) AND (acute kidney injury OR AKI OR renal dysfunction) AND (English[Language]). For inclusion, studies were required to meet the following criteria: (1) case–control, retrospective or prospective cohort, or descriptive studies published in English; (2) human subjects with laboratory-confirmed or clinically diagnosed COVID-19; and (3) reported data on pharmacological RAAS blockade exposure either before or during hospitalization and the incidence of AKI. The timing of the initiation of RAAS blockade use relative to the hospitalization date was not specified prior to analysis. Review articles, case reports, or case series reporting fewer than 10 patients or non-peer-reviewed publications were excluded. Any duplicate data among the included studies were identified by assessing whether specific studies were conducted in the same hospitals in overlapping time frames or by contacting investigators when deemed appropriate. Study selection and data extraction were conducted by two investigators (S.A.L. and R.P.) in tandem, and any discrepancies were resolved via a third reviewer’s (J.H.Y.) mediation. Although this study’s protocol was not registered in PROSPERO, no relevant protocols for this topic were found in this database during the writing of this review. Of note, the authors did not seek specific institutional review board or ethics committee approval for this study because the study was conducted using publicly available data obtained from online databases.

### Study quality and primary end points

Study quality was assessed using the Newcastle–Ottawa Scale (NOS) for retrospective studies. The NOS scale demonstrated good study quality (7–8) in all selected studies (Supplemental Table 1). The primary endpoint was the odds ratio (OR) derived from a 2 × 2 contingency table using an outcome variable equal to incident AKI and an exposure variable equal to pharmacological RAAS blockade use in COVID-19 patients. The following additional analyses were performed: an analysis of the association between incident AKI and class of RAAS blockade drug (ACEI or ARB) and an analysis of the association between RAAS blockade use and incidence of moderate-to-severe AKI.

### Data extraction and statistical analysis

The author, type of study, location where the study was performed, date of publication, median/mean age, number of AKI cases, RAAS blockade use, definition of AKI, and diagnosis criteria of COVID-19 were extracted from each included study. Meta-analysis was performed using the package ‘meta’ and ‘metafor’ of the R-project (version 3.6.0, URL https://www.R-project.org). The summary estimate of the incidence of AKI depending on RAAS blockade exposure is presented as ORs and their 95% confidence intervals (CIs). Additional analysis of the incidence of AKI depending on ACEIs/ARB use and AKI severity were performed in the same way. The pooled ORs with 95% CIs were calculated using the random-effects model with the restricted maximum-likelihood estimator assuming high heterogeneity in characteristics among the included studies. Between-study heterogeneity was assessed using the I^2^ statistic and Cochran’s Q test with *P* < 0.10. Additional univariate meta-regression was performed to find potential covariates affecting the association between RAAS blockade use and COVID-19-related AKI which can also be the source of high heterogeneity. The meta-regression included clinical factors such as age, sex, region, level of care, underlying comorbidities, and sample size. Contour-enhanced funnel plot and Egger’s test were used to detect publication bias. We also performed trim-and-fill method to evaluate the effect of missing studies and recalculated pooled OR with 95% CIs that included the hypothetical studies if deemed appropriate.

## Results

### Study selection

**Figure 1:**
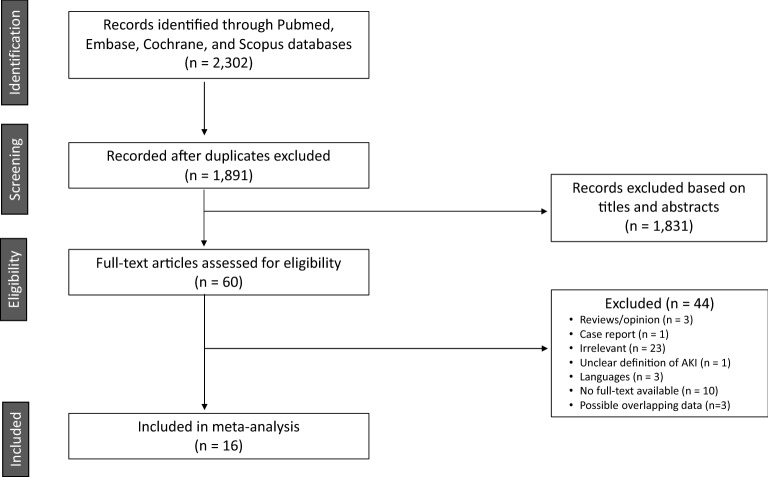
Flow diagram of the study selection.

Following the initial database search and screening, 60 full-text articles were assessed for eligibility; among them, 44 studies were excluded for specific reasons (Fig. 1). Of the remaining 16 studies, only 14 (n = 17,876) were included in the main meta-analysis that evaluated the association between RAAS blockade use and the incidence of COVID-19-related AKI. Of note, studies by Cheng et al*.* and Hirsch et al*.* were excluded because of overlapping data. However, in the sub-analysis of the association between RAAS blockade use and incident moderate-to-severe AKI, studies by Cheng et al*.* and Hirsch et al*.* were included instead of their corresponding overlapping studies (Peng et al*.* and Ng et al*.*) that did not contain the data of interest.

### Study characteristics

Table 1 shows the baseline characteristics of the included studies. Overall, the study sample sizes ranged from 23 to 9657, and mean/median ages ranged from 59 to 72 years. All included studies were observational studies performed in retrospective (n = 15) and prospective (n = 1) settings on COVID-19 patients requiring hospitalization. Studies were conducted in USA (n = 5), China (n = 2), France (n = 2), the UK (n = 2), South Korea (n = 1), Italy (n = 1), Germany (n = 1), Bahrain (n = 1), and Iran (n = 1). Among these studies reporting on incident AKI and RAAS blockade use in COVID-19, six studies reported additional RAAS blockade data stratified by drug class (ACEI vs. ARB). In addition, four studies reported additional data on AKI severity (mild vs. moderate-severe). Therefore, these studies were reviewed separately for further analysis. Hirsch et al*.* was excluded in the additional meta-analysis regarding the incidence of AKI depending on ACEIs/ARB use due to overlapping data with Ng et al*.* All studies used the Kidney Disease: Improving Global Outcomes (KDIGO) guideline for the definition of AKI. Furthermore, RAAS blockade exposure was determined based on the medication history at the time of admission in most studies; however, Cheng et al. and Kolhe et al*.* defined the exposure as drug administration during hospitalization. The criteria for COVID-19 diagnosis were based on laboratory confirmation using polymerase chain reaction in 14 studies and laboratory confirmation or clinical suspicion of COVID-19 in two studies (Soleimani et al. and Zahid et al.).

**Table 1:** Characteristics of included studies.

Author	Location	Study design	Median/mean age (IQR/SD)	Number of patients	Timing of RAAS exposure	Definition of AKI	Definition of COVID-19 infection
Chaudhri et al.^35^	New York, USA	Retrospective cohort study	59 ± 18	300	Before admission	Based on KDIGO criteria	Laboratory confirmation
Cheng et al.^36^	Wuhan, China	Retrospective cohort study	70 (59–77)	119	During hospitalization	Based on KDIGO guideline	Laboratory confirmation
Dudoignon et al.^16^	Paris, France	Retrospective cohort study	63 (57–69)	51	Before admission	Based on KDIGO criteria	Laboratory confirmation
Hirsch et al.^37^	New York, USA	Retrospective cohort study	64 (52–75)	5449	Before admission	Based on KDIGO criteria	Laboratory confirmation
Husain-Syed et al.^38^	Giessen, Germany	Prospective cohort study	60 (37–88)*	23	Before admission	Based on KDIGO criteria	Laboratory confirmation
Kolhe et al.^39^	Derby, UK	Retrospective cohort study	72^#^	1161	During hospitalization	Based on modified KDIGO criteria as identified by NHS England’s algorithm^40^	Laboratory confirmation
Lim et al.^41^	Daegu, South Korea	Retrospective cohort study	67 (57–78)	130	Before admission	Based on KDIGO criteria	Laboratory confirmation
Louis et al.^42^	Region Grand Est, France	Retrospective cohort study	N/A	181	Before admission	Based on KDIGO criteria	Laboratory confirmation
Ng et al.^43^	New York, USA	Retrospective cohort study	65^#^	9657	Before admission	Based on KDIGO criteria	Laboratory confirmation
Pelayo et al.^44^	Pennsylvania, USA	Retrospective cohort study	66 ± 15	223	Before admission	Based on KDIGO criteria except urine volume-based criterion of < 0.5 ml/kg/h for 6 h	Laboratory confirmation
Peng et al.^45^	Wuhan, China	Retrospective cohort study	61 (50–69)	4020	Before admission	Based on KDIGO criteria	Laboratory confirmation
Russo et al.^46^	Genoa, Italy	Retrospective cohort study	70 ± 16	777	Before admission	Based on KDIGO criteria	Laboratory confirmation
Soleimani et al.^18^	Tehran, Iran	Retrospective cohort study	66 ± 13	254	Before admission	Based on KDIGO criteria	Laboratory confirmation or clinical suspicion
Taher et al.^47^	Manama, Bahrain	Retrospective cohort study	54 ± 14	73	Before admission	Based on KDIGO criteria	Laboratory confirmation
Tetlow et al.^48^	London, UK	Retrospective cohort study	68 ± 17	557	Before admission	Based on KDIGO criteria	Laboratory confirmation
Zahid et al.^9^	New York, USA	Retrospective cohort study	66 (55–75)	469	Before admission	Based on KDIGO criteria	Laboratory confirmation or clinical suspicion

*Age was expressed as median [minimum–maximum] values.

^#^The mean age was re-calculated based on the separately reported mean age of each subgroup (AKI and non-AKI group).

*IQR* interquartile range, *SD* standard deviation, *RAAS* renin–angiotensin–aldosterone system, *AKI* acute kidney injury, *KDIGO* Kidney Disease: Improving Global Outcomes, *NHS* National Health Services.

### Use of RAAS blockade is associated with incident AKI in COVID-19 patients

**Figure 2:**
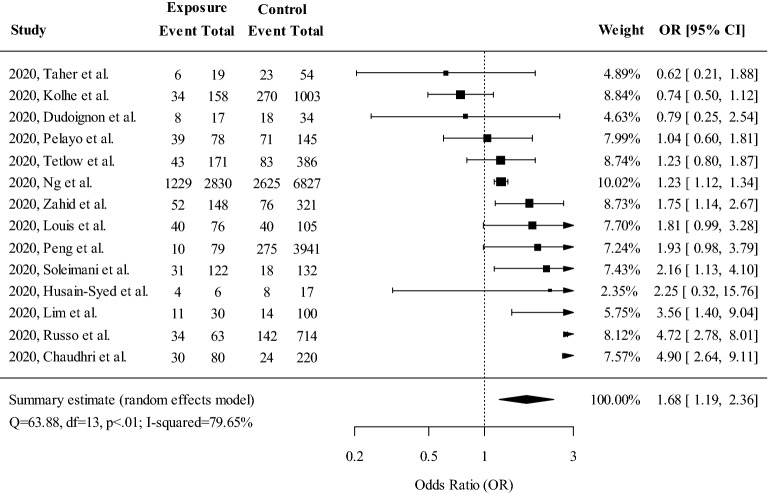
Meta-analysis of odds ratios for incident AKI in hospitalized COVID-19 patients based on exposure to RAAS inhibitors. Higher odds ratio indicates a higher risk of AKI in the RAAS blockade exposure group. *AKI* acute kidney injury, *RAAS* renin–angiotensin–aldosterone system, *OR* odds ratio, *CI* confidence interval.

**Figure 3:**
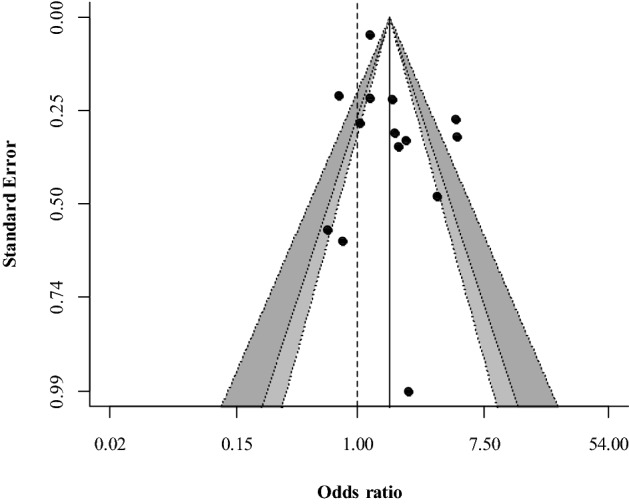
Contour-enhanced funnel plot. The symmetry of the funnel plot indicated no obvious publication bias. Inner white zone indicates P-value > 0.1, gray zone indicates 0.05 < P-value < 0.1, dark gray zone indicates 0.01 < P-value < 0.05, and outer white zone indicates P-value < 0.01.

The random-effect meta-analysis of pooled OR showed that RAAS blockade use was significantly associated with an increased risk of incident AKI in COVID-19 patients (OR 1.68; 95% CI 1.19–2.36, *P* = 0.003) with high heterogeneity (79.65%) (Fig. 2). Egger’s test was not significant, indicating no obvious publication bias (Fig. 3). Univariate meta-regression did not reveal any covariates associated with significant effect size differences (Table 2).

**Table 2:** Univariate meta-regression of possible sources of heterogeneity across the included studies.

Heterogeneity factors	Co-efficient (95% CI)	*P*-value
Mean age	1.00 (0.92, 1.10)	0.93
Male (%)	0.99 (0.98, 1.00)	0.15
**Location**		
Asia	Reference	
USA	0.72 (0.26, 1.94)	0.51
Europe	1.00 (0.42, 2.42)	1.00
**Level of care**		
Intensive care only vs. all hospitalization (reference)	0.85 (0.35, 2.03)	0.71
Hypertension (%)	0.99 (0.97, 1.01)	0.42
Diabetes (%)	0.99 (0.96, 1.01)	0.20
Chronic kidney disease (%)	0.97 (0.90, 1.05)	0.43
**Sample size**		
< 1000	Reference	
≥ 1000	0.61 (0.29, 1.29)	0.20

### Association of RAAS blockade use with incident AKI remains significant in further analyses stratified by drug class and AKI severity in hospitalized COVID-19 patients

Further sub-analysis was performed to assess whether ACEIs and ARBs had discriminative association with AKI incidence in COVID-19 patients. The random-effect meta-analysis of pooled OR showed that both ACEI use and ARB use were significantly associated with an increased risk of COVID-19-related AKI incidence (ACEI: OR 2.32; 95% CI 1.16–4.65; *P* = 0.02; ARB: OR 2.45; 95% CI 1.35–4.44; *P* = 0.003 (Supplemental Fig. 1A,B). Heterogeneity was high in both ACEI and ARB exposure analyses. Potential publication bias was found for the association between ACEI use and incident AKI through Egger’s test (Supplemental Fig. 2A). Trim-and-fill analysis implied two missing studies, and adjusted summary estimate of OR by trim-and-fill method was 1.38 with 95% CI of 0.64–3.18 (*P* = 0.5), substantially weakening the significant association found in the original analysis. No significant publication bias was found on the association between ARB use and incidence of COVID-19-related AKI based on Egger’s test (Supplemental Fig. 2B). Trim-and-fill analysis did not suggest any missing study.

Next, the association between RAAS blockade use and COVID-19-related AKI was analyzed after stratification of AKI by severity. The random-effect meta-analysis of pooled OR showed that the use of RAAS blockade was significantly associated with moderate-to-severe AKI compared to no/mild AKIin hospitalized COVID-19 patients (OR 1.31; 95% CI 1.14–1.50; *P* < 0.001) (Supplemental Fig. 3A). Low heterogeneity was found. Egger’s test on the association between RAAS blockade use and moderate-to-severe AKI was not significant, demonstrating no obvious publication bias (Supplemental Fig. 3B). Trim-and-fill analysis showed that the small study publication bias did not significantly affect the effect size (Adjusted OR 1.32; 95% CI 1.15–1.51, *P* < 0.001).

## Discussion

In this meta-analysis of published retrospective and prospective cohort studies, we observed that the exposure to RAAS blockade was associated with a higher risk of incident AKI in patients with laboratory-confirmed or clinically diagnosed COVID-19. Further meta-analyses stratified by drug class showed that the use of ARBs is associated with COVID-19-related AKI incidence, even though the association between ACEI use and COVID-19-related AKI is limited due to potential publication bias. The association between RAAS blockade use and incidence of moderate-to-severe AKI compared to those of no/mild AKI in COVID-19 patients remained significant.

ACE2 plays a seminal role in COVID-19 pathogenesis as well as RAAS pathway regulation that are critical to kidney function^19^. The binding of the coronavirus spike protein to a membrane-bound ACE2 receptor is requisite for cell invasion and ultimately for disease manifestation^20,21^. ACE2 expression is known to be significantly increased in COVID-19 patients^22^. These findings have led to a prompt therapeutic attention over whether modulation of the ACE2 activity/expression can change the clinical course of COVID-19 and whether the use of RAAS blockade will cause any difference in clinical outcome based on its modulating role of ACE2 expression/activity^23^. Several meta-analyses so far have shown that RAAS blockade use has no detrimental effect on the clinical course of COVID-19^24–26^, but the clinical evidence regarding the use of RAAS blockade on renal complication in COVID-19 is sparse.

RAAS blockade is considered nephroprotective in the long term. However, these medications can negatively affect renal function through their effects on periglomerular hemodynamics, systemic blood pressure, and natriuresis, especially in the acute setting of physiological stress^27^. Therefore, cessation of RAAS blockade use is widely applied in medical practice in the context of AKI. Based on the poorer clinical outcome of COVID-19 patients with AKI and the direct biological role of RAAS blockade in renal filtration, the potential effect of RAAS inhibitors on the incidence of AKI in COVID-19 patients is a very intriguing clinical question. To the best of our knowledge, this is the first meta-analysis on the association of recent RAAS blockade use with development of incident AKI in COVID-19 patients. Our study suggests that more careful clinical attention should be paid to COVID-19 patients with recent exposure to RAAS blockade due to their higher risk of AKI.

Even though our meta-analysis showed a significant association between recent RAAS blockade use and the incidence of AKI in COVID-19 patients, the effect of RAAS blockade use on the outcome of AKI in COVID-19 patients remains unclear. The recent randomized controlled trial comparing the clinical impact of continuation vs. discontinuation of RAAS blockades in COVID-19 patients found that there was no between-group difference in the development of acute kidney failure requiring hemodialysis^28^. Previous studies on AKI suggested continuing RAAS blockade use to prevent AKI to CKD transition based on its vaso-protective effects and mortality benefit^29,30^. Hines et al*.* showed that RAAS blockade exposure during AKI did not interrupt renal recovery following AKI^31^. The role of RAAS inhibition can be even more substantial in COVID-19-related AKI than AKI from other etiologies based on the significance of ACE2 in the pathogenesis of SARS-CoV-2 infection. Although ACE2 plays a critical role in SARS-CoV-2 viral entry, several experts have suggested that ACE2 might mediate an organo-protective role by negatively regulating the RAAS system, mediating vasodilatory, anti-inflammatory, and anti-fibrotic roles^32^. This regulatory mechanism of ACE2 is shared by RAAS inhibitors, reducing the risk of cardiovascular disease due to hypertension and diabetes. Even though our study showed a significant association between RAAS blockade use and incident AKI in COVID-19 patients, this should be carefully interpreted. Without strong pathophysiological correlation and clinical data, we should not exacerbate the hypothetical risk of RAAS blockade use in COVID-19 patients and its potential effect on incident AKI^33^.

Interestingly, our study showed a significant association between ARB use and COVID-19-related AKI incidence whereas the association of ACEI use and incident AKI in COVID-19 patients was uncertain due to significant publication bias. Further studies to understand whether there is any potential discriminative effect of ARB compared to that of ACEI on COVID-19-related AKI would be recommended based on their different site of action^34^. In addition, our meta-regression analysis did not reveal any clinical factor that can induce effect size differences on the association between RAAS blockade use and AKI incidence in COVID-19 patients. This result suggests more direct association between RAAS blockade use and COVID-19-related AKI rather than being mediated by other clinical factors like age or underlying comorbidities. Further experimental and clinical studies will be necessary to delineate the potential effect of RAAS blockade use on COVID-19-related AKI.

Our study has several limitations. A limited number of studies remained after the initial screening because most published studies regarding incident AKI with SARS-CoV-2 infection lacked specific data on recent RAAS blockade usage. Moreover, most included studies are observational retrospective studies and could only provide crude odds ratio on AKI incidence according to RAAS blockade exposure. Therefore, this meta-analysis may not be used to derive any conclusions regarding the cause-and-effect relationship between RAAS blockade and AKI in COVID-19 patients, and may also be affected by recall, selection, and publication bias. Inherent to the nature of the meta-analysis, significant heterogeneity was found both via statistical methods (I^2^, Q) as well as via individual manuscript review. This could have originated from variations in the dosage/timing of RAAS blockade use as well as different definitions of COVID-19 diagnosis, even though most of the studies followed molecular diagnosis for COVID-19 confirmation. To better understand the origin of substantial heterogeneity between studies, we performed univariate meta-regression, which did not show any significant association with effect sizes. However, other covariates such as race, the dosage of drugs, or frequency of cardiovascular disease could not be included in the meta-regression due to missing values in included studies. Multiple meta-regression could not be performed due to multiple missing covariate data among the included studies. Lastly, subgroup analyses on each potential confounding factor were limited because most of the studies simply described the overall prevalence of each factor in the total study population while no specific data was provided depending on the RAAS blockade exposure, which remains a potential weakness of our study.

In conclusion, exposure to RAAS inhibitors appears to be associated with a higher risk of COVID-19-related AKI. Because of the reliance of our analysis on observational studies, a causal relationship cannot be ascribed to RAAS blockade and AKI in COVID-19 and practice-changing recommendations cannot be made on the basis of our data. Nonetheless, our study warrants further randomized controlled studies and meta-analyses on the effect of RAAS blockade on kidney outcomes to better define the risk/benefit of RAAS blockade use in AKI patients with COVID-19.

## Supplementary Information

Supplementary Information.
