# Supplementary material for: Increased risk of acute kidney injury in coronavirus disease patients with renin–angiotensin–aldosterone-system blockade use: a systematic review and meta-analysis
Source: Sci Rep. 2021 Jun 30;11:13588. doi: 10.1038/s41598-021-92323-8 (PMC8245570; doi:10.1038/s41598-021-92323-8)
Supplement: Supplementary file 1 — Supplementary Information. [file 41598_2021_92323_MOESM1_ESM.pdf]

Increased risk of acute kidney injury in coronavirus disease patients with renin-angiotensin-aldosterone-system blockade use: A systematic review and meta-analysis

Sul A Lee, M.D.[1,2], Robin Park, M.D.[2], Ji Hyun Yang, M.D.[2], In Kyung Min, M.S.[3], Jung Tak Park, M.D., Ph.D.[1], Seung Hyeok Han, M.D., Ph.D.[1], Shin-Wook Kang, M.D., Ph.D.[1], Tae-Hyun Yoo, M.D., Ph.D.[1*]

Affiliations:

[1] Department of Internal Medicine, College of Medicine, Institute of Kidney Disease Research, Yonsei University, Seoul, South Korea

[2] Department of Medicine, MetroWest Medical Center/Tufts University School of Medicine, Framingham, MA, U.S.A.

3 Biostatistics Collaboration Unit, Department of Biomedical Systems Informatics, Yonsei University College of Medicine, Seoul, South Korea

Supplemental Table 1. Newcastle-Ottawa Scale

	Selection 1)	Selection 2)	Selection 3)	Selection 4)	Comparability 1)	Outcome 1)	Outcome 2)	Outcome 3)	Score
Chaudhri *et al.*[1]	b	a	a	a	a	a	a	a	8
Cheng *et al.*[2]	b	a	a	a	a	a	a	a	8
Dudoignon *et al.*[3]	b	a	d	a	a	a	a	a	7
Hirsch *et al.*[4]	b	a	a	a	a	a	a	a	8
Husain-Syed *et al.*[5]	a	a	a	a	a	a	a	a	8
Kolhe *et al.*[6]	b	a	a	a	a	a	a	a	8
Lim *et al.*[7]	b	a	b	a	a	a	a	a	8
Louis *et al.*[8]	b	a	d	a	a	a	a	a	7
Ng *et al.*[9]	a	a	a	a	a	a	a	a	8
Pelayo *et al.*[10]	b	a	d	a	a	a	a	a	7
Peng *et al.*[11]	a	a	a	a	a	a	a	a	8
Russo *et al.*[12]	b	a	a	a	a	a	a	a	8
Soleimani *et al.*[13]	b	a	a	a	a	a	a	a	8
Taher *et al.*[14]	b	a	a	a	a	a	a	a	8
Tetlow *et al.*[15]	b	a	a	a	a	a	a	a	8
Zahid *et al.*[16]	b	a	a	a	a	a	a	a	8

Reference

1. Chaudhri, I. *et al.* Outcomes Associated with the Use of Renin-Angiotensin-Aldosterone System Blockade in Hospitalized Patients with SARS-CoV-2 Infection. *Kidney360* **1**, 801 (2020).
2. Cheng, Y. *et al.* Risk Factors and Outcomes of Acute Kidney Injury in Critically Ill Patients with Coronavirus Disease 2019. *Kidney Diseases* (2020).
3. Dudoignon, E. *et al.* Activation of the renin-angiotensin-aldosterone system is associated with Acute Kidney Injury in COVID-19. *Anaesth Crit Care Pain Med* **39**, 453-455 (2020).
4. Hirsch, J. S. *et al.* Acute kidney injury in patients hospitalized with COVID-19. *Kidney Int* **98**, 209-218 (2020).
5. Husain-Syed, F. *et al.* Acute kidney injury and urinary biomarkers in hospitalized patients with coronavirus disease-2019. *Nephrol Dial Transplant* **35**, 1271-1274 (2020).
6. Kolhe, N. V., Fluck, R. J., Selby, N. M. & Taal, M. W. Acute kidney injury associated with COVID-19: A retrospective cohort study. *PLoS Med* **17**, e1003406 (2020).
7. Lim, J. H. *et al.* Adverse impact of renin-angiotensin system blockade on the clinical course in hospitalized patients with severe COVID-19: a retrospective cohort study. *Sci Rep* **10**, 20250 (2020).
8. Louis, G., Belveyre, T., Goetz, C., Gaci, R. & Dinot, V. Acute kidney injury in severe SARS-CoV-2 infection: An experience report in Eastern France. *Anaesth Crit Care Pain Med* **40**, 100788 (2020).
9. Ng, J. H. *et al.* Outcomes Among Patients Hospitalized With COVID-19 and Acute Kidney Injury. *Am J Kidney Dis* (2020).
10. Pelayo, J. *et al.* Clinical Characteristics and Outcomes of Community- and Hospital-Acquired Acute Kidney Injury with COVID-19 in a US Inner City Hospital System. *Cardiorenal Med* **10**, 223-231 (2020).
11. Peng, S. *et al.* Early versus late acute kidney injury among patients with COVID-19-a multicenter study from Wuhan, China. *Nephrol Dial Transplant* **35**, 2095-2102 (2020).
12. Russo, E. *et al.* Kidney disease and all-cause mortality in patients with COVID-19 hospitalized in Genoa, Northern Italy. *J Nephrol*, 1-11 (2020).
13. Soleimani, A. *et al.* Effects of angiotensin receptor blockers (ARBs) on in-hospital outcomes of patients with hypertension and confirmed or clinically suspected COVID-19. *Am J Hypertens* (2020).
14. Taher, A., Alalwan, A. A., Naser, N., Alsegai, O. & Alaradi, A. Acute Kidney Injury in COVID-19 Pneumonia: A Single-Center Experience in Bahrain. *Cureus* **12**, e9693 (2020).
15. Tetlow, S. *et al.* ACE inhibitors, angiotensin receptor blockers and endothelial injury in COVID-19. *J Intern Med* (2020).
16. Zahid, U. *et al.* Acute Kidney Injury in COVID-19 Patients: An Inner City Hospital Experience and Policy Implications. *Am J Nephrol* **51**, 786-796 (2020).

PRISMA 2009 Checklist

Supplemental Table 2. PRISMA 2009 Checklist

Section/topic	#	Checklist item	Reported on page #
**TITLE**			
Title	1	Identify the report as a systematic review, meta-analysis, or both.	1
**ABSTRACT**			
Structured summary	2	Provide a structured summary including, as applicable: background; objectives; data sources; study eligibility criteria, participants, and interventions; study appraisal and synthesis methods; results; limitations; conclusions and implications of key findings; systematic review registration number.	3-4
**INTRODUCTION**			
Rationale	3	Describe the rationale for the review in the context of what is already known.	5-6
Objectives	4	Provide an explicit statement of questions being addressed with reference to participants, interventions, comparisons, outcomes, and study design (PICOS).	5-6
**METHODS**			
Protocol and registration	5	Indicate if a review protocol exists, if and where it can be accessed (e.g., Web address), and, if available, provide registration information including registration number.	7
Eligibility criteria	6	Specify study characteristics (e.g., PICOS, length of follow-up) and report characteristics (e.g., years considered, language, publication status) used as criteria for eligibility, giving rationale.	7
Information sources	7	Describe all information sources (e.g., databases with dates of coverage, contact with study authors to identify additional studies) in the search and date last searched.	7
Search	8	Present full electronic search strategy for at least one database, including any limits used, such that it could be repeated.	7
Study selection	9	State the process for selecting studies (i.e., screening, eligibility, included in systematic review, and, if applicable, included in the meta-analysis).	7
Data collection process	10	Describe method of data extraction from reports (e.g., piloted forms, independently, in duplicate) and any processes for obtaining and confirming data from investigators.	8
Data items	11	List and define all variables for which data were sought (e.g., PICOS, funding sources) and any assumptions and simplifications made.	8
Risk of bias in individual studies	12	Describe methods used for assessing risk of bias of individual studies (including specification of whether this was done at the study or outcome level), and how this information is to be used in any data synthesis.	8-9
Summary measures	13	State the principal summary measures (e.g., risk ratio, difference in means).	7-8
Synthesis of results	14	Describe the methods of handling data and combining results of studies, if done, including measures of consistency (e.g., I^2) for each meta-analysis.	8

PRISMA 2009 Checklist

Section/topic	#	Checklist item	Reported on page #
Risk of bias across studies	15	Specify any assessment of risk of bias that may affect the cumulative evidence (e.g., publication bias, selective reporting within studies).	8
Additional analyses	16	Describe methods of additional analyses (e.g., sensitivity or subgroup analyses, meta-regression), if done, indicating which were pre-specified.	8-9
**RESULTS**			
Study selection	17	Give numbers of studies screened, assessed for eligibility, and included in the review, with reasons for exclusions at each stage, ideally with a flow diagram.	10
Study characteristics	18	For each study, present characteristics for which data were extracted (e.g., study size, PICOS, follow-up period) and provide the citations.	10-11
Risk of bias within studies	19	Present data on risk of bias of each study and, if available, any outcome level assessment (see item 12).	10-12
Results of individual studies	20	For all outcomes considered (benefits or harms), present, for each study: (a) simple summary data for each intervention group (b) effect estimates and confidence intervals, ideally with a forest plot.	11-12, Fig.2, Supp. Fig. 1 & 3
Synthesis of results	21	Present results of each meta-analysis done, including confidence intervals and measures of consistency.	11-12
Risk of bias across studies	22	Present results of any assessment of risk of bias across studies (see Item 15).	10-12
Additional analysis	23	Give results of additional analyses, if done (e.g., sensitivity or subgroup analyses, meta-regression [see Item 16]).	11-12
**DISCUSSION**			
Summary of evidence	24	Summarize the main findings including the strength of evidence for each main outcome; consider their relevance to key groups (e.g., healthcare providers, users, and policy makers).	13
Limitations	25	Discuss limitations at study and outcome level (e.g., risk of bias), and at review-level (e.g., incomplete retrieval of identified research, reporting bias).	15-16
Conclusions	26	Provide a general interpretation of the results in the context of other evidence, and implications for future research.	16
**FUNDING**			
Funding	27	Describe sources of funding for the systematic review and other support (e.g., supply of data); role of funders for the systematic review.	20

From: Moher D, Liberati A, Tetzlaff J, Altman DG, The PRISMA Group (2009). Preferred Reporting Items for Systematic Reviews and Meta-Analyses: The PRISMA Statement. PLoS Med 6(7): e1000097. doi:10.1371/journal.pmed1000097

For more information, visit: **www.prisma-statement.org**.

A

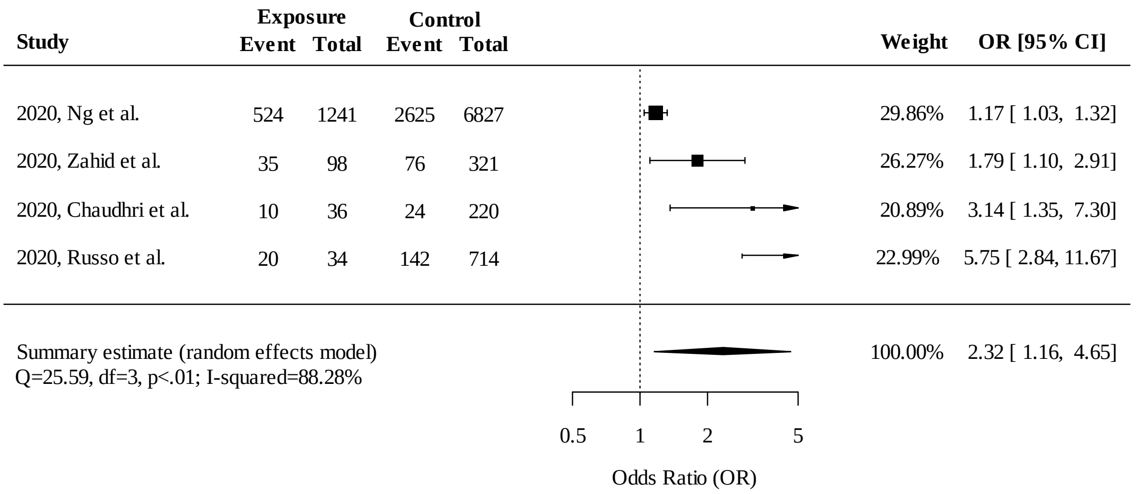

B

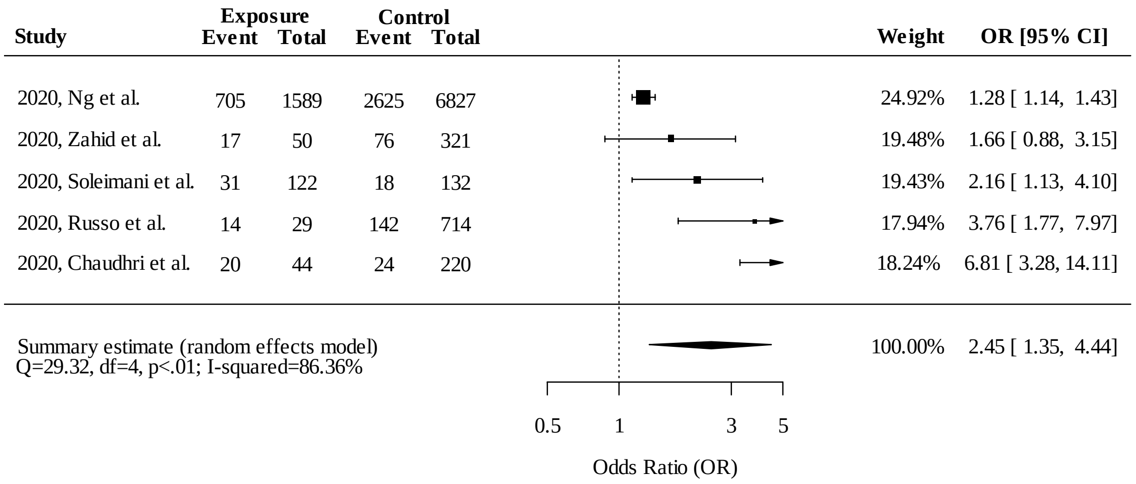

Supplemental Figure 1. Meta-analysis of pooled odds ratios for incident AKI in hospitalized COVID-19 patients based on exposure to ACEIs or ARBs

Significant association was found between COVID-19-related AKI incidence and recent exposure to (A) ACEIs as well as (B) ARBs.

Abbreviations: AKI, acute kidney injury; ACEI, angiotensin-converting-enzyme inhibitor; ARB, angiotensin receptor blocker; OR, odds ratio; CI, confidence interval.

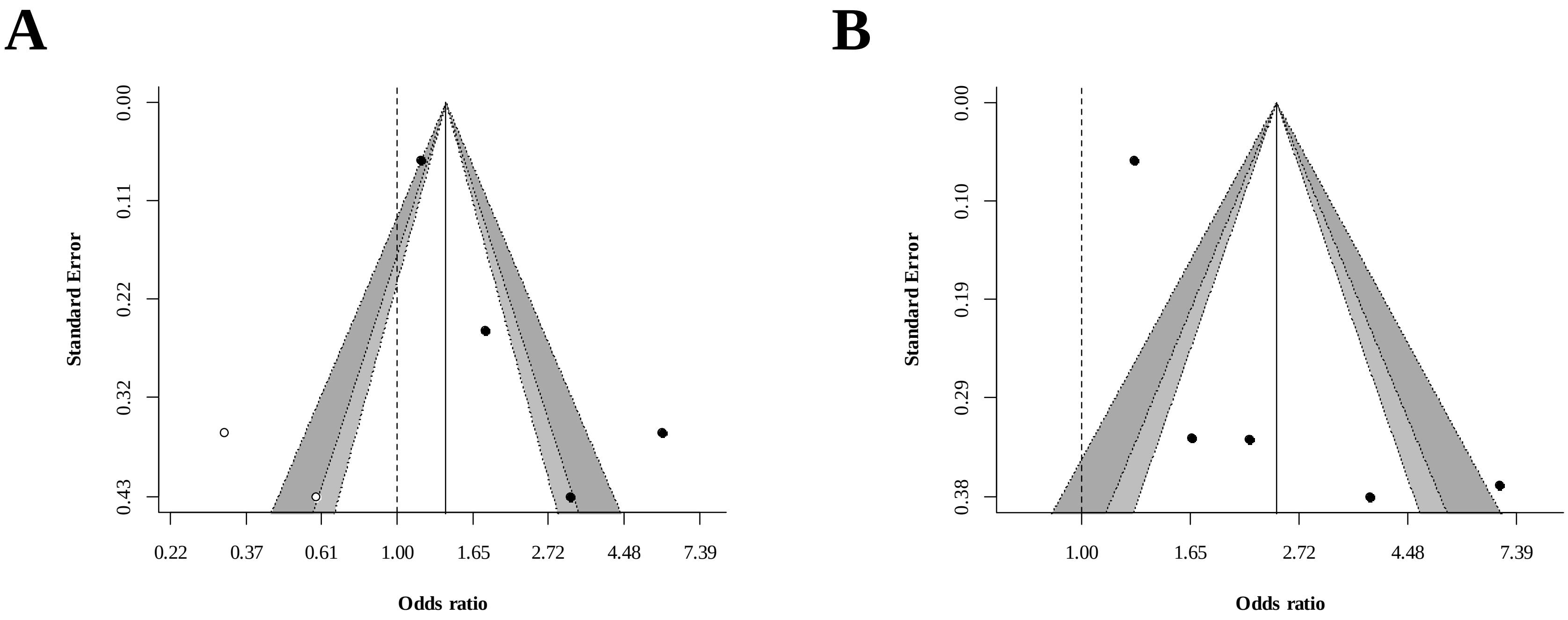

Supplemental Figure 2. Contour-enhanced funnel plot of meta-analysis of the association between ACEI/ARB use and incident AKI in COVID-19 patients

(A) The funnel plot indicates potential publication bias in the meta-analysis regarding the association between ACEI use and COVID-19-related AKI. (B) No publication bias was found in the meta-analysis of the association of ARB use with COVID-19-related AKI. Inner white zone indicates p-value > 0.1, gray zone indicates 0.05 < p-value < 0.1, dark gray zone indicates 0.01 < p-value < 0.05, and outer white zone indicates p-value < 0.01.

Abbreviations: ACEI, angiotensin-converting-enzyme inhibitor; ARB, angiotensin receptor blocker; AKI, acute kidney injury

A

Study	Exposure Event	Exposure Total	Control Event	Control Total	Weight	OR [95% CI]
2020, Cheng et al.	0	6	34	113	0.22%	0.18 [0.01, 3.23]
2020, Taher et al.	5	19	16	54	1.35%	0.85 [0.26, 2.75]
2020, Hirsch et al.	352	1556	714	3893	90.50%	1.30 [1.13, 1.50]
2020, Zahid et al.	34	148	51	321	7.93%	1.58 [0.97, 2.57]
Summary estimate (random effects model) Q=2.92, df=3, p=0.40					100.00%	1.31 [1.14, 1.50]

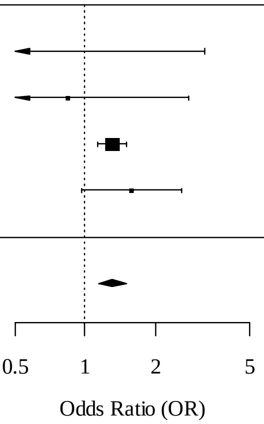

B

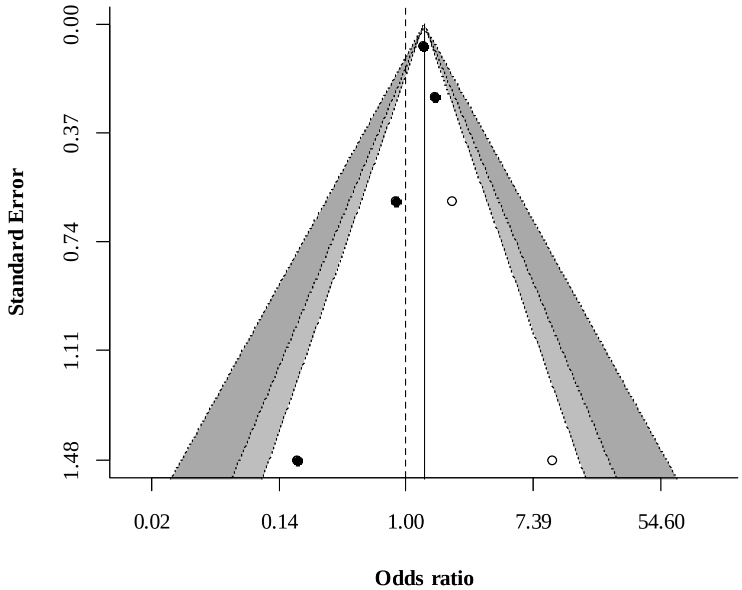

Supplemental Figure 3. Meta-analysis of pooled odds ratios for moderate-to-severe vs. no/mild AKI in hospitalized COVID-19 patients based on RAAS blockade use

(A) RAAS blockade exposure was significantly associated with the incidence of moderate-to-severe AKI compared to no/mild AKI in hospitalized COVID-19 patients. (B) The funnel plot indicates no potential publication bias in the association of RAAS blockade use on incident moderate-to-severe AKI compared to no/mild AKI in COVID-19 patients requiring hospitalization. Inner white zone indicates p-value > 0.1, gray zone indicates 0.05 < p-value < 0.1, dark gray zone indicates 0.01 < p-value < 0.05, and outer white zone indicates p-value < 0.01

Abbreviations: RAAS, renin-angiotensin-aldosterone-system; AKI, acute kidney injury
